# A single muscle moves a crustacean limb joint rhythmically by acting against a spring containing resilin

**DOI:** 10.1186/1741-7007-7-27

**Published:** 2009-05-29

**Authors:** Malcolm Burrows

**Affiliations:** 1 Department of Zoology, University of Cambridge, Cambridge, UK

## Abstract

**Background:**

The beating or fanning movements of three pairs of maxilliped flagella in crabs and crayfish modify exhalent gill currents while drawing water over chemoreceptors on the head. They play an integral part both in signalling by distributing urine odours, and in active chemosensation.

**Results:**

The rhythmical maxilliped movements start with maxilliped 3 followed after a delay of 15 to 20 ms in shore crabs by maxilliped 2 and then maxilliped 1, at a frequency of 18 to 20 Hz in crabs and 10 to 13 Hz in signal crayfish. The contraction of a single abductor muscle controls the power stroke (abduction) of each flagellum, which is accompanied by flaring of feather-like setae which increase its surface area. No muscle can bring about the return stroke (adduction). Release of an isolated flagellum from an imposed abduction is followed by a rapid recoil to its resting adducted position. The relationship between the extent of abduction and the angular velocity of the return stroke indicates the operation of a spring. Blue fluorescence under UV light, and its dependence on the pH of the bathing medium, indicates that resilin is present at the joint between an exopodite and flagellum, at the annuli of a flagellum and at the base of the setae.

**Conclusion:**

Resilin is progressively bent as a flagellum is abducted and resumes its natural shape when the joint recoils. Other distortions of the exopodites may also contribute to this spring-like action. The joint is therefore controlled by a single abductor muscle operating against a spring in which the elastic properties of resilin play a key role.

## Background

The actions of sets of limb muscles are constrained by the mechanics of the joints they move in animals with either an internal or an external skeleton. Where the mechanical arrangement of a joint allows movement in only a single plane, a pair of muscles is usually present which can act antagonistically so that one muscle is responsible for effecting movement in one direction and a second for movement in the opposite direction. At joints where there is greater freedom of movement, more muscles are present and different movements result from different combinations of actions of the whole muscle set, or a subset of the muscles. Across the animal kingdom, however, there are some joints where only one muscle is present, implying that there must be some restorative force to move these joints in the opposite direction.

In vertebrates, the tensor tympani alone acts on the malleus bone and pulls it medially so tensing the tympanic membrane and dampening the oscillations in the ossicular chain. In bivalve molluscs such as mussels and scallops, a large adductor muscle closes the two shells, which then re-open due to the elasticity imparted to the hinge joint between them by the presence of the rubber-like protein abductin [1,2]. In spiders, only a flexor muscle is present at certain limb joints; this acts against the hydrostatic pressure of the blood, which is controlled by the actions of distant muscles, and which brings about extension when the flexor relaxes [3]. In many arachnids the hydrostatic forces may be aided by elastic recoil of sclerites in the articular membrane [4]. In scorpions, the single muscle that straightens the tibio-tarsal joint works against the bending forces of the body mass [5]. In insects, a single retractor unguis muscle curls the most distal segment of the tarsus and works against elastic fibres and the elasticity of the joint itself [6,7]. The aperture of some insect spiracles, which allow an exchange of air between the inside and outside of the body, is reduced by a single closer muscle and increased by elastic recoil of the two spiracular valves due to the elasticity of a cuticular bridge [8].

In crustaceans, the joint between the ischus and merus of a walking leg has only a flexor muscle [9] and is said in a review [10] to operate against a pad-like ligament containing the elastic protein resilin [11], which brings about extension. Resilin, an almost perfect elastic, is found in many places in insects and in some crustaceans where energy must be stored, or where rapid and full recoil is needed. For example, it occurs in the tendons of dragonfly flight muscles [11], in the tymbal of cicadas [12] and in the jumping mechanisms of fleas [13] and froghoppers [14]. Resilin is highly deformable and shows almost perfect elastic recovery [10]. For example, when the tendon of the pleuro-alar muscle of the dragonfly *Aeshna *was stretched to twice its length for long periods, it returned to its original length once the load was removed [10]. Resilin can act as a useful spring over a wide range of speeds, as it loses less than 5% of its energy during movements in which it is deformed at rates as high as 200 Hz [15]. Resilin has two key signatures that enable it to be recognised [10,14,16-19]; first it fluoresces in the blue when illuminated by a narrow band of near ultraviolet (UV) light and, second, the fluorescence is reversibly dependent on pH.

This investigation analyses the operational consequences of moving a joint with just one muscle by focusing on a particular joint in the maxillipeds of crabs and crayfish. In each of these three pairs of mouthpart appendages, the joint between the exopodite and flagellum has a single abductor muscle. The rhythmical contractions of these abductor muscles cause the flagella to beat in a co-ordinated sequence at frequencies of 7 to 16 Hz depending upon the species [20]. Their beating or fanning, in which they act as rakes or leaky paddles [21], creates flow fields that can deflect the exhalent water current after it has passed over the gills. The beating can also draw water from a wide area in front of the animal [22] over chemosensory neurons on the antennules and mouthparts [23]. The flow created in this way mixes odour molecules in the water and thus enables better odour acquisition and sampling than by ambient flow or molecular diffusion alone [24]. The antennules flick regularly and are swept downwards to enhance the penetration of odours to the receptor cells [25,26]. The beating movements of the maxillipeds may also allow an assessment of the amount of particulate matter in the exhalent current that might indicate the need for cleaning the gills [27]. Finally, the currents created by the beating may be responsible for the controlled distribution of odour molecules released in the urine from nearby nephropores and used in signalling to conspecifics [28-30].

This investigation shows that the power stroke of each beating movement of a maxilliped flagellum is caused by active contractions of a single abductor muscle, but that the return stroke is brought about by the elastic recoil of a spring containing resilin.

## Methods

Crabs, *Carcinus maenas *(Crustacea, class Malacostraca, order Decapoda, suborder Pleocyemata, infraorder Brachyura, family Portunidae) were caught at Wells-next-the Sea, Norfolk, UK, and *Cancer productus *at Friday Harbor, Washington, USA. Crayfish *Pacifastacus leniusculus *(infraorder Astacida, family Astacidae) a species recently invasive in the UK from the USA, were taken from freshwater at Histon, Cambridge, UK. The sizes of the crabs are expressed as the width of the carapace at its widest point, and of the crayfish as the length of their body from rostrum to tail.

Sequential images of the natural beating movements of the three pairs of maxillipeds of restrained crabs and crayfish were captured at rates of 1000 s^-1 ^and exposure times of 0.17 to 0.33 ms with a Photron Fastcam 1024PCI high speed camera (Photron (Europe) Ltd, Marlow, Bucks., UK). Images of passive adduction movements following imposed abductions of excised individual maxillipeds were captured at rates of 250 s^-1 ^and with an exposure time of 0.05 ms. The images were fed directly to a laptop computer. Two movies of these movements (Movie 1 from the crab *Carcinus*, and Movie 2 from a crayfish) are included as Additional files 1 and 2. Selected image files were analysed with Motionscope camera software (Redlake Imaging, San Diego, CA, USA) to determine the duration and motion of each cycle of movement of the flagella of the maxillipeds. The angular motions of the flagella were measured from individual frames imported into Canvas X (ACD Systems of America, Miami, FL, USA).

Electrical recordings were made from the flagella abductor muscles of the maxillipeds of five *Cancer *during natural beating with pairs of implanted 75 μm diameter copper wires insulated but for their tips. The resulting signals were conditioned with AC coupled amplifiers and then displayed and photographed from an oscilloscope screen.

To search for the presence of resilin, maxillipeds of *Carcinus *and *Pacifastacus *were placed in their respective saline or in 50% glycerol in a Petri dish with a floor of Sylgard™ on the stage of an Olympus BX51WI compound microscope. They were viewed through Olympus MPlan 5×/0.1 NA, MPlan10×/0.25 NA and LUCPlanFLN 20×/0.45 NA objective lenses, under UV or white epi-illumination. UV light was provided by an X-Cite 120 metal halide light source conditioned by a DAPI-5060B Brightline series (Semrock, Rochester, NY, USA) high-brightness UV filter set with a sharp-edged passband from 350 nm to 407 nm (1% transmission limits). The resulting blue fluorescence emission was collected in a similarly sharp-edged band at wavelengths from 413 nm to 483 nm through a dichromatic beam splitter. Epi- illumination in the visible range was provided by flexible light guides, one on each side of the fixed stage of the microscope, attached to a Schott KL1500 light source. Images were captured with a Nikon DXM1200 digital camera as colour (RGB) TIFF files. During each experiment, the camera gain and exposure time were kept fixed. Images captured at the same focal planes under UV and visible light were superimposed in Canvas X.

The movements and structure of the maxilliped joints were analysed in 17 crabs and 11 crayfish at water temperatures of 17 to 21°C.

## Results

### Organisation of the maxillipeds

**Figure 1:**
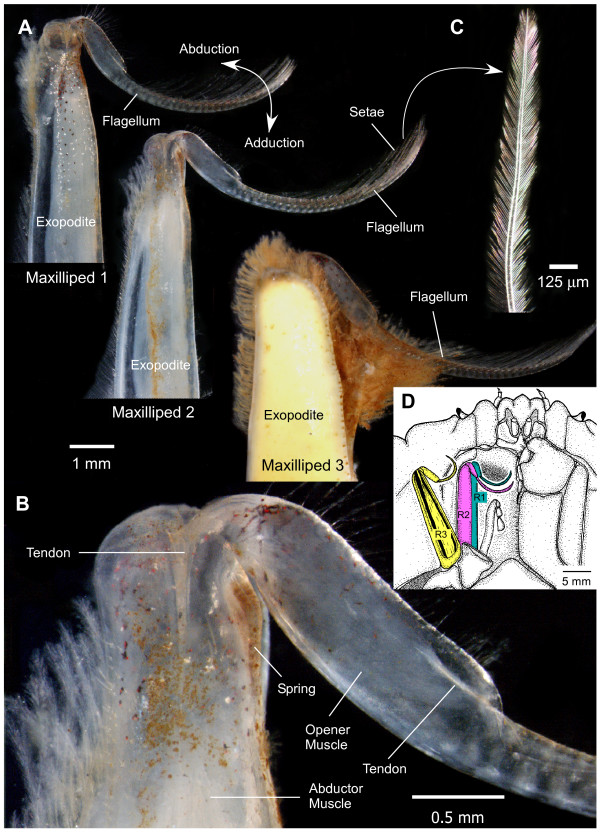
**Photographs of maxillipeds in the crab *Carcinus***. **(A) **Views of the ventral (outside) surfaces of the exopodites and flagella of the three right maxillipeds. Scale bar 1 mm. **(B) **Higher power view of the outside of the joint between the exopodite and flagellum of maxilliped 2. Scale bar 0.5 mm. **(C) **A plumose seta from the flagellum of maxilliped 2. Scale bar 125 μm. **(D) **Drawing of the maxillipeds in relation to the oral cavity and other appendages. The same colour coding of the maxillipeds is used in Figures 2 and 3. Scale bar 5 mm.

The three sets of maxillipeds on each side are arranged to form a pair of stacks at the exhalent opening of the ventilatory current from the gills, close to the mouth (Figure 1A to 1D). In crabs, the endopodites of the third pair of maxillipeds meet at the midline and by rotation at their bases can widen or restrict the aperture of the chamber around the mouth. The exopodites of the third maxillipeds are the most ventral and in crabs their outer surfaces are calcified and hard (Figure 1A). The exopodites of the second and first maxillipeds lie directly inside this and are made of more flexible and transparent cuticle (Figure 1A). Each endopodite contains a single abductor muscle that moves an annulated flagellum. The muscles are numbered 78 in maxilliped 1, 87 in maxilliped 2 and 102 in maxilliped 3 according to the scheme proposed by [9]. In a crab with a carapace width of 43 mm, the three flagella ranged in length from 5 to 5.5 mm and each had a mass of 0.7 mg. In a crayfish 118 mm long, the flagella of maxillipeds 1 to 3 ranged from 6 to 7 mm in length and had masses of 1.7 mg, 2.6 mg and 2.95 mg respectively.

In its resting position a flagellum was naturally curved (Figure 1A). In crabs, the resting position was almost fully adducted, so that the base of a flagellum lay against the medial surface of its exopodite, but in crayfish the resting position was midway between full adduction and full abduction. The most proximal segment of a flagellum is enlarged in crabs and contains a small opener muscle (numbered 79, 86, 103 in, respectively, maxillipeds 1, 2 and 3 [9]) (Figure 1B). Contraction of one of these muscles bends the distal segments of its flagellum in the same direction as the abduction of the whole flagellum while at the same time it is said to flare the two rows of plumose setae [31]. Each segment (annulus) of a flagellum has a pair of these setae that are about 1.5 mm long, and in turn each seta has two rows of finer hairs about 100 μm long (Figure 1C). In crabs these hairs are densely packed about 10 μm apart. At rest the setae lay almost parallel to the long axis of a flagellum with their tips pointing to its distal end and when flared the fine hairs of adjacent setae interlaced with each other to form an extensive sieve. The net result was to increase the effective surface area of each flagellum as it was actively moved through the water by contractions of the abductor muscle in its exopodite.

### Beating movements of the maxillipeds

**Figure 2:**
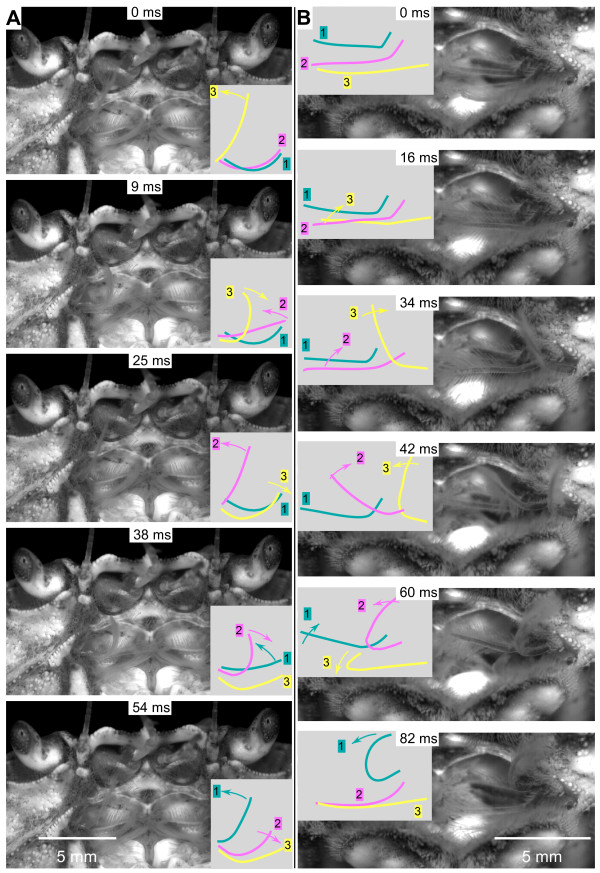
**Natural beating movements of the flagella of the three pairs of maxillipeds in the crab *Carcinus *restrained under water**. Individual frames from sequences captured at 1000 s^-1 ^are arranged in two columns, with each sequence starting with a power stroke (abduction) movement of maxilliped 3. The panels accompanying each frame are drawings of the positions of each flagellum (colour coded as in Figure 1) with the arrows indicating their direction of movement. **(A) **The camera pointed into the oral cavity of a female crab with a carapace width of 45 mm. The eyecups, antennules and antennae are visible at the top. **(B) **A closer view of the oral cavity of a second female crab (carapace width 61 mm) viewed from a more ventral perspective. Scale bars 5 mm.

**Figure 3:**
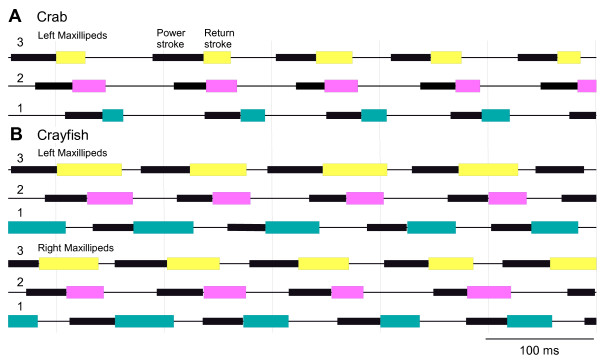
**Sequence of movements by the maxilliped flagella**. A power stroke is indicated by the thick black bars and the return stroke (adduction) by coloured boxes (following the same colour coding as in Figures 1 and 2) and intervening periods of inactivity are represented by the thin black lines. **(A) **Crab, *Carcinus*. The maxillipeds on one side move with the power strokes occurring in the sequence 3, 2 and 1. In the example shown, the frequency of movements was 10 Hz and thus lower than average for a crab. **(B) **Crayfish. The maxillipeds move at the same time on both sides, with the power strokes on each side following the sequence 3, 2 and 1. The power stroke in maxilliped 3 on the right side precedes that of left maxilliped 3.

The sequence of maxilliped beating, or fanning, movements on one side of the body began with the third maxilliped (Figure 2A), although in prolonged bouts of beating, this maxilliped sometimes dropped out. The flagellar setae were flared to a position where they subtended an angle of 45 to 55° so that the flagellum presented its greatest surface area when it was actively swept through the water during abduction (Figure 2B). It is not known to what extent the flaring was caused by the resistance of the water as the flagellum was moved, or by contraction of the small opener muscle which has processes extending into each annulus of the flagellum. At the start of the return stroke (adduction), the setae moved to a position parallel to the long axis of the flagellum so that they offered less resistance. The return stroke of the setae must either result from the resistance of the water or some spring-like mechanism as there is no muscle that could move them in this direction. The same sequence of actions was repeated after a delay of 15 to 25 ms by maxilliped 2 and then after a similar delay by maxilliped 1 (Figures 2A, 2B and 3A). The whole cycle of movements by the three maxillipeds on one side could then be repeated for long periods, or was succeeded by a switch to movements of the maxillipeds on the other side. At other times the maxillipeds on both sides did not move.

In crabs the duration of the power stroke for maxilliped 3 was 33 ms (mean of means from three crabs, standard deviation for all 38 cycles of movements measured was ± 5.6), for maxilliped 2 it was 30.7 ± 6.7 ms (103 cycles) and for maxilliped 1 it was 29.1 ± 3.6 ms (112 cycles). The return stroke in crabs was always faster (24 ± 5.0, 23.2 ± 4.0 and 20.7 ± 3.4 ms respectively in maxillipeds 3, 2 and 1) so that this phase occupied about 42% of the cycle time of one complete beat. By contrast, in crayfish the duration of the power stroke was 46 ms (mean of means from four crayfish, standard deviation for all 62 cycles of movements by each of the three maxillipeds was ± 5.6), and the return stroke was 49.2 ± 13.1 ms, so that this phase occupied about 52% of the cycle time of one complete beat. In three crabs the mean frequency of the flagellar beating ranged from 18 to 20 Hz and in four crayfish, the mean frequency was lower, at 10 to 13 Hz.

In the crab *Carcinus *only the maxillipeds on one side moved in this sequence at any one time though occasional beats on the two sides sometimes overlapped as beating switched from one side to the other. By contrast, in the crayfish *Pacifastacus *the maxillipeds on both sides were often moved at the same time in a complex but co-ordinated and repeatable pattern (Figure 3B). On one side, the power strokes of the maxilliped movements followed the same sequence of 3, 2 and 1 as in crabs but with longer delays of 30 to 40 ms between each maxilliped. The two sides were co-ordinated in such a way that the power stroke of a particular maxilliped on one side preceded that on the other; in the example shown in Figure 3 the right side preceded the left. A particular maxilliped sometimes failed to contribute to the rhythm for one or more cycles without disturbing the subsequent pattern. Maxilliped 3 was the most frequent to drop out.

### Pattern of muscle activity in the maxillipeds

**Figure 4:**
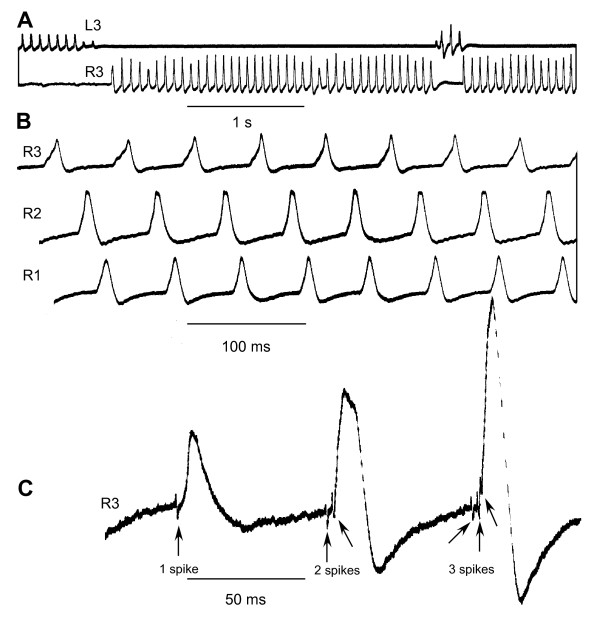
**Recordings of electrical activity from the maxilliped abductor muscles of the crab *Cancer *during natural beating movements**. **(A) **The rhythmic beating of the left (L3) and right (R3) maxillipeds alternates. Each compound junctional potential in the muscle corresponds to one beat of a flagellum. **(B) **Sequence of action of the three maxillipeds on one side, which starts with a contraction of the muscle of maxilliped 3 (R3) and is followed by maxilliped 2 (R2) and then by maxilliped 1 (R1). **(C) **A recording from the abductor muscle in which an increasing number of spikes in the motor neurons preceded the progressively larger compound muscle potentials.

The pattern of motor impulses to the abductor muscles underlies the overall pattern of the maxilliped movements (Figure 4). Recordings from the third maxillipeds on the left and right sides of a crab showed alternating activity; a repetitive sequence of muscle potentials on one side was replaced by the same rhythm on the other side (Figure 4A) or silence ensued on both sides. Each compound potential in the muscle was associated with an abduction movement of a flagellum. Recordings from the three maxillipeds on one side showed a consistent pattern that began with a compound potential in maxilliped 3, followed after a delay of 15 to 25 ms by a compound potential in maxilliped 2, and then after a similar delay by one in maxilliped 1 (Figure 4B).

The amplitude of the potentials was usually consistent but some were markedly smaller than the average. In some recordings it was possible to discern the more rapid spikes of the motor neurons that caused the longer-lasting muscle potentials (Figure 4C). Between one and three motor spikes occurred at each cycle of the movement, with the number of spikes positively correlated with the amplitude of the compound muscle potential. The occasional smaller muscle potentials are thus explained by fewer motor spikes. The potentials caused by successive spikes within one cycle summed and sometimes evoked an active response from the muscle membrane. All the electrical activity recorded from an exopodite was associated with the abduction phase of the movement and none was observed that could be ascribed to the return phase of the cycle. What therefore brings about the return (adduction) phase?

### Spring-like recoil of the maxilliped flagella

Exopodites of each maxilliped were removed from crabs and crayfish and secured in dishes of saline with a floor of Sylgard in such a way that their flagella could move freely. The flagella all adopted their natural resting posture and because they were separated from the nervous system could not be moved actively by any muscular contractions. The setae did not flare during these imposed movements, suggesting that the opener muscle must play an active part in bringing about flaring during natural beating movements. Finally, the isolation of the maxillipeds meant that active changes in blood pressure could not contribute to any of the movements.

**Figure 5:**
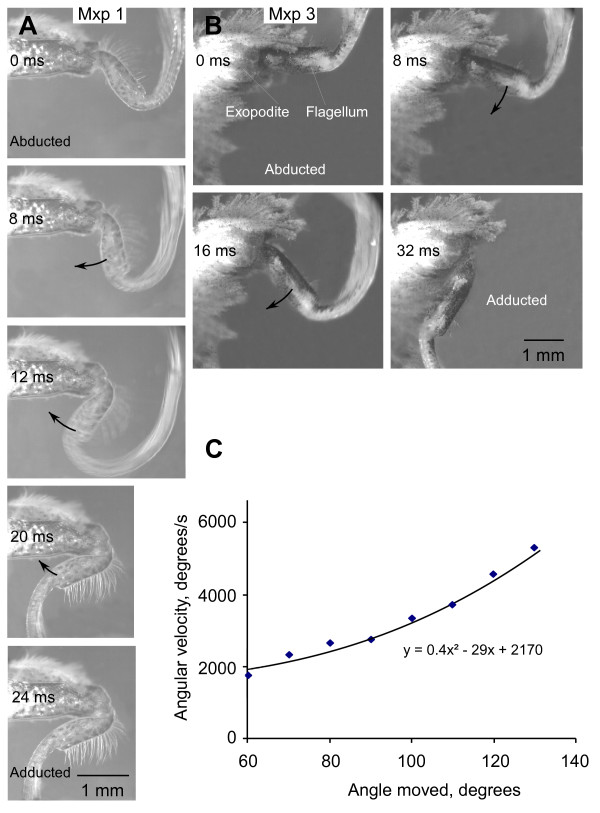
**Elastic recoil of a flagellum in the crab *Carcinus *after an imposed abduction movement**. **(A) **Maxilliped 1. **(B) **Maxilliped 3. Each flagellum was abducted by imposed movement and then released. Selected frames captured at the times indicated after release are shown from sequences captured at 250 images s^-1^. **(C) **Graph of the average angular velocity of the recoil movement against the angular extent of abduction. The graph is based on 28 imposed movements of maxilliped 3, 16 of maxilliped 2 and 11 of maxilliped 1 from one male and two female crabs. The average velocity for all maxillipeds is plotted for 10° bins of angles moved. Scale bar in A and B 1 mm.

A high-speed camera captured images at rates of 250 s^-1 ^as a flagellum was forcibly abducted and then released, whereupon it recoiled rapidly to its resting position (Figures 5A and 5B). In crabs the recoil from full, forced abduction lasted on average 50% longer than the return stroke during natural beating, probably because they were abducted through a greater angle. The average angular velocity over the complete recoil movement depended on the extent to which a flagellum had initially been forcibly abducted (Figure 5C). In three crabs, the flagella of maxillipeds 1 to 3 were each forcibly abducted through different angles and then released. The greater the angle through which a flagellum was abducted the greater was the average angular velocity of the recoil movement, indicating the presence of a spring.

**Figure 6:**
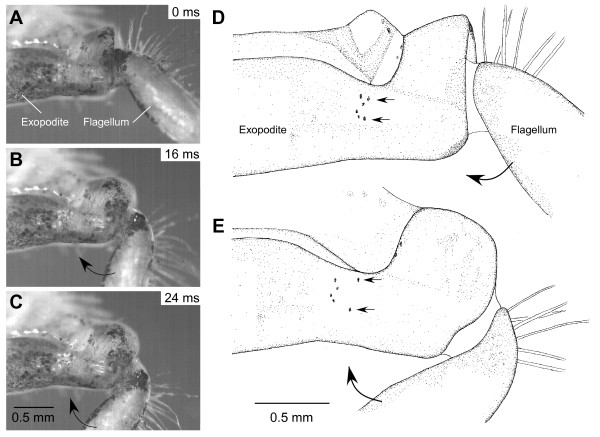
**Distortions of the exopodites of maxilliped 2 of the crab *Carcinus *during abduction**. **(A, B, C) **Three photographs taken at the times indicated from a sequence captured at 250 images s^-1 ^as the flagellum recoiled from a forced abduction. Scale bar 0.5 mm. **(D, E) **Drawings of the maxilliped at its most abducted and adducted positions. The small horizontal arrows point to dark spots on the exopodite which mark the distortions that occur during the recoil from abduction to adduction. Scale bar 0.5 mm.

During forced abductions, the inner maxillipeds 1 and 2 showed clear distortions of the cuticle of their exopodites, which recovered during the recoil to adduction (Figure 6). These distortions were to the ventral and distal edges of an exopodite (Figure 6A) and to a dorsal area just proximal to the joint with the flagellum (Figure 6B). They were particularly apparent when the pattern of pigmented spots on the medial surface of the distal exopodite was compared in the fully abducted and adducted positions (two horizontal arrows in Figure 6D and 6E). For example, the two spots indicated by the arrows moved closer together during abduction and then moved apart by more than 100 μm during the recoil movement.

### Fluorescence at the joint between an exopodite and a flagellum

The operation of a spring bringing about the return stroke (adduction) prompted a search for where energy could be stored during contraction of the abductor muscle and then released to power the recoil. A potential candidate is the protein resilin which has the requisite elastic properties and can be recognised by the specific properties of its bright blue fluorescence when illuminated with particular wavelengths of UV light (see Methods).

**Figure 7:**
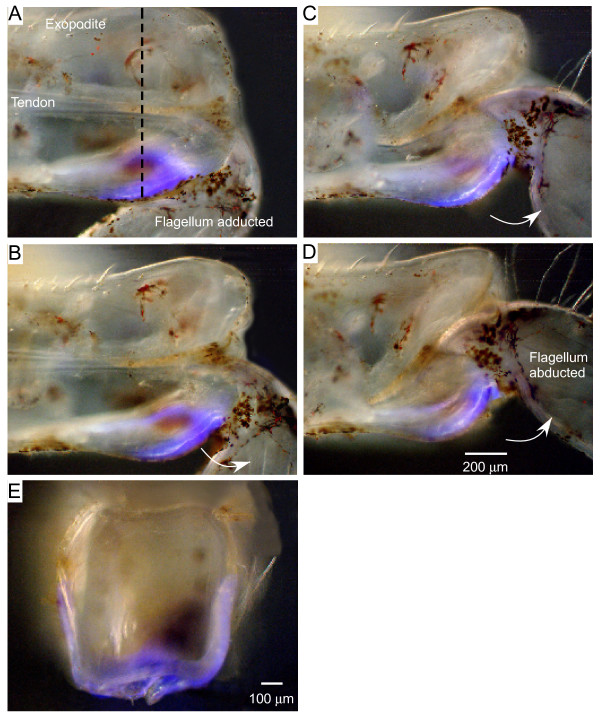
**Superimposed images of maxilliped 2 of the crab *Carcinus *viewed ventrally under bright field and UV illumination**. The ventral (outside) cuticle and underlying hypodermis of the exopodite were removed. **(A) **With the flagellum at its natural resting position (adducted), a strip of bright blue fluorescence on the ventral surface of the joint is almost straight. **(B, C, D) **As the flagellum is progressively abducted the fluorescent strip becomes progressively bent. **(E) **Section through the exopodite at the plane indicated by the dashed line in A shows the fluorescence along the bottom surface and wrapping around the ventral and dorsal surfaces. Scale bars A, B, C, D 200 μm, E 100 μm.

Under such UV illumination, elongated strips of bright blue fluorescence about 400 μm long and 100 to 200 μm wide and 50 to 100 μm thick were revealed along the bottom edge of both the ventral and dorsal surfaces of the distal exopodites of crab maxillipeds and their facing edges (Figure 7). In the resting, fully adducted position of a crab flagellum, these strips of resilin were almost straight (Figure 7A). As the flagellum was forcibly abducted through increasingly greater angles the strips were progressively bent (Figures 7B and 7C) so that at full abduction they were bent at right angles to the bottom edge of the exopodite (Figure 7D). Upon release from the imposed abduction, the fluorescent strips returned rapidly to their original straight shape at full adduction. Sections through the joint showed that the fluorescence was present on the bottom surface of the exopodite and wrapped around both its ventral and dorsal surfaces (Figure 7E) thus approximating a U-girder in cross section.

**Figure 8:**
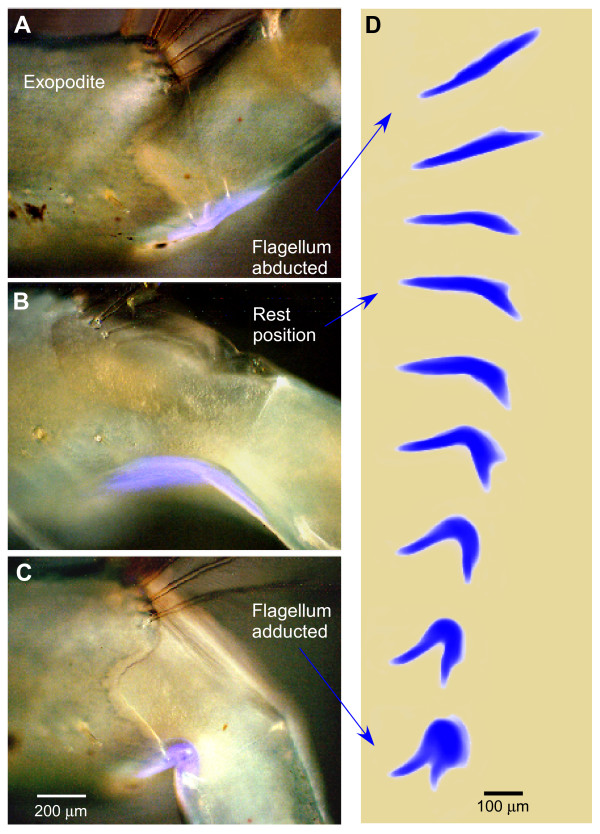
**Fluorescence at the joint between the exopodite and flagellum of crayfish maxilliped 2**. **(A, B, C) **The ventral strip of fluorescence at three positions of the flagellum about the exopodite. Bright field and UV images are superimposed. **(A) **Fully abducted. **(B) **Natural resting position. **(C) **Adducted. **(D) **The shape of the fluorescence at nine different angles of the same flagellum about its exopodite, from full abduction to full adduction. Only the UV images are shown. Scale bars A, B, C 200 μm, D 100 μm.

In crayfish, the resting position of a flagellum, when there is no contraction by the abductor muscle, was mid way between full abduction and adduction at an angle of about 110° (Figure 8). At this position the strips of fluorescence were slightly curved (Figures 8A and 8B). When the flagellum was abducted the curved resilin strips were straightened (Figure 8A), and when the flagellum recoiled beyond, or was forced past, its resting position to a more adducted position, then the strips were bent into an acute angle (Figures 8C and 8D). The extent to which the fluorescent strips were bent during flagellar movements about an exopodite was clearly seen when its shape was displayed at these different angles (Figure 8D).

### Fluorescence at joints in a flagellum

The annulated flagellum itself also bends and its setae are spread laterally during natural beating movements, but again only one muscle is present that could affect these movements. Contractions of the opener muscle in the base of the flagellum could bend the flagellum at the annuli and could spread the setae. What, however, causes the flagellum to resume its natural resting shape and the setae to close and lie parallel to the long axis of the flagellum?

**Figure 9:**
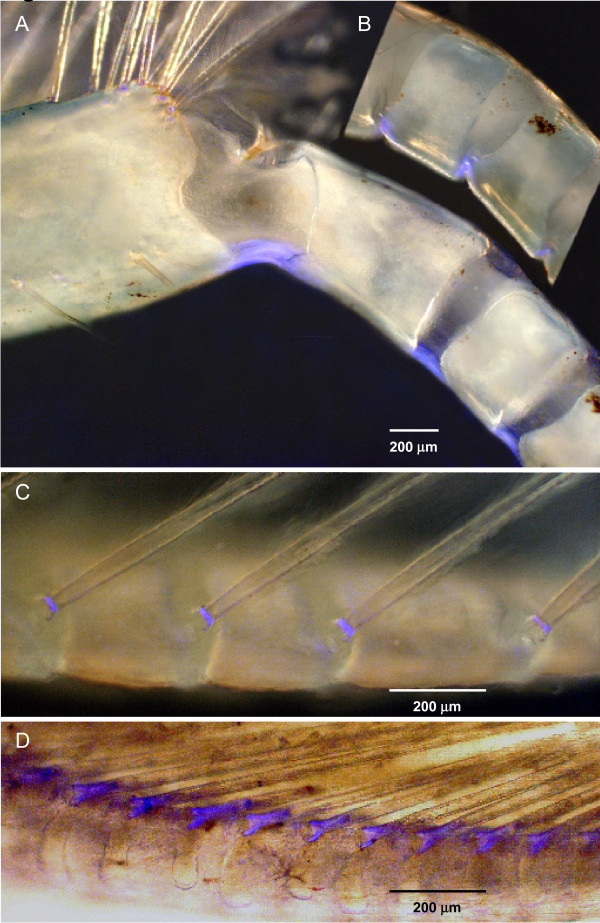
**Fluorescence at joints in a flagellum**. **(A) **Patches of fluorescence at joints between the proximal annuli of the flagellum of a crayfish maxilliped 2 at their normal resting position. **(B) **The same patches distorted when the curvature of the flagellum was forcibly decreased. **(C) **Setae on the flagellum of maxilliped 2 of a crayfish. Fluorescence is restricted to a ring at the base of each seta. **(D) **Flagellum of maxilliped 2 in a crab *Carcinus *to show the fluorescence at the base of each seta. Scale bars 200 μm.

Illumination of the flagella with UV light revealed the presence of a short strip of resilin on the inside surface between each annulus (Figure 9A). When the flagellum was forcibly bent, as occurred during the natural cycle of beating, the resilin was also bent (Figure 9B), but then resumed its natural shape when the force was removed and the flagellum itself returned to its normal shape. These strips of resilin at the joints between the annuli therefore appear to act in the same way as those at the joints between an exopodite and a flagellum. Resilin was also present at the base of each of the setae. In crayfish the resilin formed a ring around the base of a seta (Figure 9C), but in crabs had a more elaborate structure with two arms forming the pivots in a socket on the flagellum, and a broader region extending into the shaft of the seta (Figure 9D).

### Properties of the blue fluorescence

The intense blue fluorescence was only visible when viewed with emission filters that transmitted wavelengths from 413 nm to 483 nm. With other emission filters (for example, CY3 and GFP) no fluorescence was detected. The emission spectrum of the fluorescence is thus the same as the first key signature of resilin.

The intensity of the blue fluorescence was sensitive to the pH of the medium bathing a maxilliped. When the pH of the sea water or saline surrounding a crab maxilliped was lowered from its neutral state to pH 3.0, the intensity of the fluorescence progressively declined so that it had almost disappeared after 10 to 14 min. When the maxilliped was returned to a neutral or alkaline medium the intensity increased again and after 20 to 25 min achieved levels seen at the start of the experiment. The fluorescence is thus reversibly sensitive to the pH of the bathing medium. This property of the fluorescence is thus the same as the second key signature for resilin.

## Discussion

The flagellum of each of the three pairs of maxillipeds in a crab or a crayfish beats rhythmically powered solely by the contractions of a single abductor muscle. In *Carcinus *the three maxillipeds on one side beat at 18 to 20 Hz in a sequence that begins with the abduction of maxilliped 3 and is followed after a delay by the abduction of maxilliped 2 and then maxilliped 1. In *Pacifastacus*, the maxillipeds on both sides can beat at the same time at 10 to 13 Hz, but still follow the same sequence. The net effect is to create water currents that both distribute the exhalent ventilatory stream from the gills in different directions, but which, at the same time, also draw water toward the oral region from the front of the animal. The return stroke of the flagellar movements is passive in both crabs and crayfish, and is brought about by the recoil of a spring that is progressively tensed during the contraction of the abductor muscle and the resulting abduction movement. The flagellum of an isolated exopodite, and therefore one that is devoid of neural or muscular control, will spring back to its normal resting position when released from a forced abduction. Bright fluorescence isolated to the blue part of the spectrum, a signature of the elastic protein resilin, is present at the joint between an exopodite and a flagellum. Resilin at this position would impart elastic recoil properties to the spring. Blue fluorescence is also present at the joints between the annuli of a flagellum, and at the base of each seta in the two lateral rows. Again only a single muscle is present in a flagellum which could change its curvature and the orientation of the setae during the power and return strokes. The unfurling of the annuli and the folding of the setae to reduce drag during the return stroke of a flagellum may thus also be controlled by biomechanical springs containing resilin.

### Is the fluorescence from resilin?

Resilin is colourless and consists of coiled peptide chains linked together in a three dimensional network by the amino acids dityrosine and trityrosine [16-18]. Dityrosine, trityrosine and resilin each emit a blue fluorescence with a maximum near 420 nm when excited by near UV light. The fluorescence described here in the maxillipeds was observed with a sharp-edged, narrow UV filter set that limited the observed emission to the 420 to 480 nm band, where most of the emission from resilin lies. The specificity of the fluorescence was apparent from its failure to be observed under different narrow band emission filters.

A defining characteristic of resilin and its amino acid links is that their fluorescence is reversibly pH-dependent, declining in acidic and increasing in alkaline pH [17]. The excitation spectrum of the acidic form of resilin lies at much shorter wavelengths than the lower limit (450 nm) used here so would not have been activated at pH 3 [14]. The fluorescence observed here also declined progressively when moved from its natural neutral saline and placed in a saline of acidic pH and recovered in a saline of alkaline pH. These two specific and diagnostic tests for the presence of resilin were therefore met in these experiments. A chemical analysis of the proteins present in the regions where the fluorescence is seen and the presence of resilin inferred would complete the identification, but no such successful analysis has yet been reported.

The correlation between the detailed fluorescence properties and the presence of resilin has been made most strongly in insects for the tendons of flight muscle [10], the tarsal pads of cockroaches [19] and for the pleural arches of froghoppers [14]. Numerous other examples have been reported where a blue fluorescence has been taken to indicate the presence of resilin; for example, in the tymbals of cicadas [12,32], as a presumptive energy storage device in fleas [13,33,34], in the veins of damselfly wings [35] and the tarsi of ants [36]. Dityrosine and trityrosine may, however, be used as protein cross-links where resilin is not present, and impart fluorescence to structures such as the chorion of *Drosophila *eggs [37]. In this paper the same optical set-up was used for the detection of fluorescence as in the analysis of the energy storage mechanisms in froghoppers and in which a rigorous series of tests was used to establish that the fluorescence was due to the presence of resilin [14]. It seems reasonable to conclude therefore that the blue fluorescence seen in the maxillipeds of crabs and crayfish is from resilin. This interpretation is also in keeping with other examples in crustaceans where blue fluorescence has been correlated with the presence of resilin. For example, in the parasitic copepod, *Pennella elegans*, the cuticle linking the anterior of the body that is embedded in the host fish and the posterior part of the body that remains outside fluoresces bright blue in UV light and this fluorescence is quenched in acid pH and enhanced in alkaline pH [38]. Furthermore both dityrosine and trityrosine are present in this region of cuticle, which also has elastic properties. The sequences of proteins extracted from the inter-segmental membranes of lobster are more closely related to the proteins from insect pliant membranes than to those in calcified cuticle [39] and may have a stiffness that is similar to that of resilin [40].

## Conclusion

The spring at the joint between an exopodite and a flagellum of a maxilliped returns the flagellum to its resting position following the contraction of the single abductor muscle. Similarly, if the flagellum of an isolated exopodite is forcibly abducted and then released it springs back to its resting position with an angular velocity that is dependent on the extent of the imposed abduction. The relationship between velocity and angular displacement for each maxilliped indicated a spring operating over the normal range of movements of a flagellum.

What constitutes the spring? Three possible components of the joint between the exopodite and a flagellum may contribute. First, the stiff tendon of the abductor muscle itself may be elastic although there is no indication that it contains resilin. Its recoil after contraction may aid the adduction. Second, the distal soft cuticle of maxilliped 2 of a crab is distorted at full abduction but returns to its natural shape at its resting position of full adduction. Again no resilin was revealed where the distortions occur although the potential to store energy here must be possible. In the harder exopodite of maxilliped 3 no comparable distortions have been observed, but the velocity of recoil is comparable to that of the other two maxillipeds. Third, the main element appears to be the elasticity of the distal region of the exopodite at the joint with the flagellum. This is distorted during natural movements of a flagellum and contains resilin.

In crabs the resting position of a flagellum is at full adduction when the fluorescent strips along the bottom edges of the ventral and dorsal surfaces of an exopodite are almost straight. Contraction of the abductor muscle then bends the resilin into almost a right angle at full abduction, but it then resumes its natural shape during the recoil during adduction. In crayfish, the resting position of a flagellum is part-way between adduction and abduction where the fluorescent strips are partially bent. Contraction of the abductor muscle causes these strips to straighten at full abduction. Conversely, if the flagellum moves closer to full adduction by a forced movement then the strips are progressively bent. In crayfish, therefore, bending the fluorescent strips in either direction away from their preferred shape results in a passive recoil movement of a flagellum back to its resting position.

The springs within a flagellum itself, in both crabs and crayfish, identified by the presence of resilin, may operate on the same principle. Bending the annuli from their preferred position bends the strip of resilin at each joint, resulting in a passive recoil back to the preferred position. Similarly the resilin at the base of the setae may help to ensure that the lateral flaring which occurs before the start of the power stroke of a flagellum is restored by passive recoil before the start of the return phase. Viscous drag may also ensure that there is hydrodynamic coupling between the fine hairs on the setae, and together with the orientation of the setae themselves must presumably also contribute to the restoration of shape during the return phase. The result is that surface area of a flagellum is increased during the power stroke and is reduced during the return stroke, or adduction, so that it offers less drag. In all of these springs, the ability of resilin to act as an almost perfect rubber and to return to its original shape without creep or energy loss is critical to its function of restoring the position of the joints rapidly and allowing the next contraction of the power stroke muscle to deliver a consistent effect.

### Why have joints with only one muscle?

The best that can be achieved at a joint with just one muscle is that the spring against which the muscle must act returns the joint to the same position after each contraction. Even to achieve this the spring must have almost perfect recoil and be able perform consistently with high repetition rates, which, for the maxilliped flagella can be as high as almost 20 Hz. The pay-off may lie in the fact that elimination of an antagonistic muscle allows an increase in the size and hence force that the power-producing muscle can produce in a limb where space is limited. There may also be a pay-off in the simplicity of the control system needed to operate such a joint. Sensory feedback might be able to control the extent of the power stroke movements of the maxillipeds, but may be able to exert little effect on the return stroke as a simple reset mechanism. Where greater flexibility is needed in the movements of a joint, the more conventional use of at least one muscle responsible for each direction of movement offers greater opportunities for control. Nevertheless, the simplicity of operation of joints with a muscle acting against a spring suggests that they may be more common than currently appears. For example, the external filament of an antennule of a crayfish which is flicked rapidly and periodically to sample the water is depressed by active contractions of a muscle, but no muscle is present that could affect the return stroke, which is thus surmised to be brought about by the elasticity of the joint [41]. In insects, phorid flies (*Iridophora clarki*) parasitise ants by rapidly injecting eggs with a spring loaded ovipositor which appears to operate by muscles acting against resilin as the spring [42].

## Supplementary Material

Additional file 1 **Movie 1**. Beating of the maxillipeds of the crab *Carcinus maenas *captured at a frame rate of 1000 s^-1 ^and exposure time of 0.33 ms. Click here for file

Additional file 2 **Movie 2**. Beating of the maxillipeds of the crayfish *Pacifastacus leniusculus *captured at a frame rate of 1000 s^-1 ^and exposure time of 0.33 ms. Click here for file
